# Calling on a million minds for community annotation in WikiProteins

**DOI:** 10.1186/gb-2008-9-5-r89

**Published:** 2008-05-28

**Authors:** Barend Mons, Michael Ashburner, Christine Chichester, Erik van Mulligen, Marc Weeber, Johan den Dunnen, Gert-Jan van Ommen, Mark Musen, Matthew Cockerill, Henning Hermjakob, Albert Mons, Abel Packer, Roberto Pacheco, Suzanna Lewis, Alfred Berkeley, William Melton, Nickolas Barris, Jimmy Wales, Gerard Meijssen, Erik Moeller, Peter Jan Roes, Katy Borner, Amos Bairoch

**Affiliations:** 1 Erasmus Medical Centre, Department of Medical Informatics, Dr. Molewaterplein 40/50, NL-3015 GE Rotterdam, the Netherlands; 2 Department of Human Genetics, Centre for Medical Systems Biology, Leiden University Medical Centre, 2300 RC Leiden NL, Einthovenweg 20, 2333 ZC Leiden, the Netherlands; 3 Knewco Inc., Fallsgrove Drive, Rockville, MD 20850, USA; 4 Open Progress Foundation, Olstgracht, 1315 BH AlmereAlmere, the Netherlands; 5 The GO consortium, EMBL-European Bioinformatics Institute, Hinxton, Cambridge, and Department of Genetics, University of Cambridge, Hinxton, CB10 1SD, United Kingdom; and Berkeley Bioinformatics Open-source Projects, Lawrence Berkeley National Laboratory, Cyclotron Road, Berkeley, CA 94720, USA; 6 Swiss Institute of Bioinformatics, Swiss-Prot Group and Department of Structural Biology and Bioinformatics, University of Geneva, CMU - Rue Michel-Servet, 1211 Genève 4, Switzerland; 7 Stanford Medical Informatics, NCBO, Campus Drive, Stanford, CA 94305-5479, USA; 8 BioMed Central, Cleveland Street, London W1T 4LB, UK; 9 EMBL - European Bioinformatics Institute, IntAct database, Hinxton, Cambridge CB10 1SD, UK; 10 SciELO, BIREME/PAHO/WHO, Rua Botucatu, 862, Vila Clementino 04023-901, São Paulo SP, Brazil; 11 Istituto Stela, Rua Prof. Ayrton Roberto de Oliveira, 32, 7° andar Itacorubi, Florianópolis-SC, 88034-050, Brazil; 12 The WikiMedia Foundation, San Francisco, CA 94107-8350, USA; 13 Indiana University, S. Indiana Ave, Bloomington, IN 47405-7000, USA

## Abstract

WikiProteins enables community annotation in a Wiki-based system. Extracts of major data sources have been fused into an editable environment that links out to the original sources. Data from community edits create automatic copies of the original data. Semantic technology captures concepts co-occurring in one sentence and thus potential factual statements. In addition, indirect associations between concepts have been calculated. We call on a 'million minds' to annotate a 'million concepts' and to collect facts from the literature with the reward of collaborative knowledge discovery. The system is available for beta testing at .

## Rationale and overview

This paper aims to explain an experimental system for community annotation and collaborative knowledge discovery called WikiProteins.

The exploding number of papers abstracted in PubMed [1,2] has prompted many attempts to capture information automatically from the literature and from primary data into a computer readable, unambiguous format. When done manually and by dedicated experts, this process is frequently referred to as 'curation'. The automated computational approach is broadly referred to as text mining. The term text mining itself is ambiguous in that it means very different things to different people [2]. In a recent debate there is a perceived controversy between pure text mining approaches to recover facts from texts and the manual curation approach [3,4]. We propose here that a combination of text mining and subsequent community annotation of relationships between concepts in a collaborative environment is the way forward [5].

The future outlook to integrate data mining (for instance gene co-expression data) with literature mining, as formulated in the review by Jensen *et al*. [2], is at the core of what we aim for at the text mining/data mining interface. To support the capturing of qualitative as well as quantitative data of different natures into a light, flexible, and dynamic ontology format, we have developed a software component called Knowlets™. The Knowlets combine multiple attributes and values for relationships between concepts.

**Figure 1:**
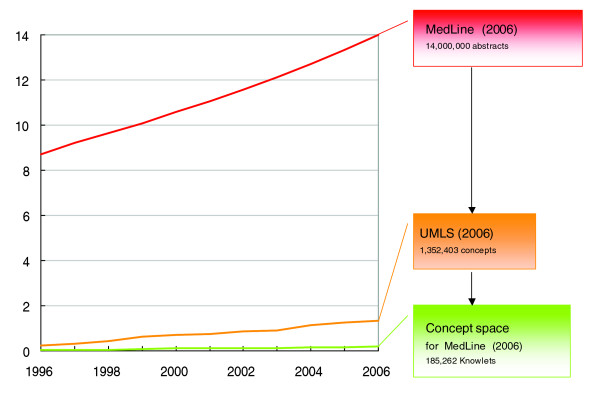
PubMed grew beyond 14,000,000 abstracts in 2006 (by the end of 2007 the 17,000,000 mark was passed). In 2006, UMLS contained well over 1,300,000 concepts. Only 185,262 concepts from UMLS were actually mentioned in PubMed (2006 version) and, therefore, the concept space of the entire PubMed corpus could be captured in just over 185,000 Knowlets.

Scientific publications contain many re-iterations of factual statements. The Knowlet records relationships between two concepts only once. The attributes and values of the relationships change based on multiple instances of factual statements (the F parameter), increasing co-occurrence (the C parameter) or associations (The A parameter). This approach results in a minimal growth of the 'concept space' as compared to the text space (Figure 1).

The first section of this article describes the WikiProteins application and rationale in general terms. The second section describes three user scenarios enabled by the current status of the Knowlet-based Wiki system. In the third section (provided as Additional data file 1) a more detailed technical description of the system is given.

## WikiProteins

WikiProteins is a web-based, interactive and semantically supported workspace based on Wiki pages and connected Knowlets of over one million biomedical concepts, selected from authorities such as the Unified Medical Language System (UMLS) [6], UniProtKB/Swiss-Prot [7] IntAct [8] and the Gene Ontology (GO) [9]. Progressively more biological databases and ontologies, such as the Genetic Association Database, can be added [10], although not all of these may have an authoritative status. The terminological data derived from these resources has been entered and mapped to unique concept identifiers in a Wiki-based terminology system called OmegaWiki [11]. More detailed information regarding biomedical concepts can be viewed in the WikiProteins user interface.

**Figure 2:**
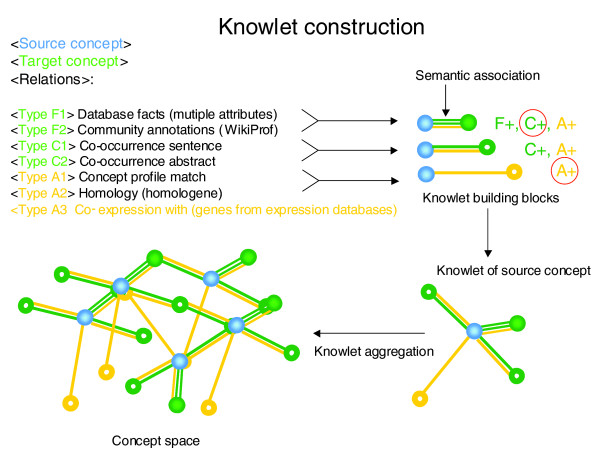
Any concept in the biomedical literature - for instance, a protein or a disease - can be treated as a source concept (depicted as a blue ball throughout the picture and the system). There may be curated information in authoritative databases such as UMLS or UniProtKB/Swiss-Prot concerning the concept and its factual relationships with other concepts. This information is captured and all concepts that have a 'factual' relationship with the source concept in any of the participating databases are thus included in the Knowlet of that concept. These 'factually associated concepts' are depicted in the Knowlet visualisation as solid green balls. In addition, the source concept may be mentioned with other concepts in one and the same sentence in the literature. In that case, especially when there are multiple sentences in which the two concepts co-occur, there is a high chance for a meaningful, sometimes causal, relationship between the two concepts. Most concepts that have a factual relationship are likely to be mentioned in one or more sentences in the literature at large, but as we have mined only PubMed so far, there might be many other factual associations that are not easy to recover from PubMed abstracts alone. For instance, many protein-protein interactions described in UniProtKB/Swiss-Prot cannot be found as co-occurrences in PubMed. Target concepts that co-occur minimally once in the same sentence as the source concept are depicted as green rings in the visualisation of the Knowlet. The last category of concepts is formed by those that have no co-occurrence per sentence in the indexed resources but have sufficient concepts in common with the source concepts in their own Knowlet to be of potential interest. These concepts are depicted as yellow rings and could represent implicit associations. Over one million Knowlets have been created so far. Each source concept has a relationship of varying strength with other (target) concepts and each of these distances has been assigned with a value for factual (F), co-occurrence (C) and associative (A) parameters. All Knowlets are dynamically coupled into the concept space. The semantic association between each concept pair is computed based on these values. In the near future additional data will be added, such as co-expression statistics between genes.

In WikiProteins each concept can be edited by the community. Each concept page is hyperlinked to the Knowlets of all concepts mentioned in that page. A Knowlet stores relationships between a given source concept and individual target concepts. The various relationships (F, C and A) between two concepts are computed into a single composite value, named the 'semantic association'. The technology allows the coupling of all Knowlets into a larger, dynamic ontology called the 'concept space' (Figure 2).

Knowlets and their connections can be exported into standard ontology and web languages such as the Resource Description Framework (RDF) and the Web Ontology Language (OWL) [12]. Therefore, any application using these languages will enable the use of Knowlet output for reasoning and querying with programmes such as the SPARQL Protocol and RDF Query Language [13]. The concept space is provided in open access. The system performs a recalculation of the semantic relationships in the entire biomedical concept space at regular intervals.

The Knowlet forms a 'related concept cloud' around a given concept, where each relationship is attributed with a semantic association with a given value. Spurious co-occurrences between concepts of specific semantic types, such as a drug and a disease or a protein and a tissue, in one and the same sentence are rare. Such co-occurrences may still occur, for instance, based on erroneous mapping of ambiguous terms to the wrong concepts. Spurious correlations can be reported and corrected by the community in WikiProteins.

Filters can be applied by users so that only associations between semantic types of their specific interest are shown. Currently, the following semantic groups are supported: anatomy, chemicals, diseases, organisms, proteins (and their genes), and a general class of 'others' (all other semantic types classified in the UMLS [6]). In addition, Knowlets can be viewed with a 'background mode' filter to mainly show factual and strong co-occurrence associations, and with a 'discovery mode' filter where more weight is given to indirect associations.

### The new Wiki component

In WikiProteins, for each source concept a unique Wiki page has been created describing the preferred thesaurus term, the synonyms, one or more definitions and the annotations as derived from authoritative databases.

**Figure 3:**
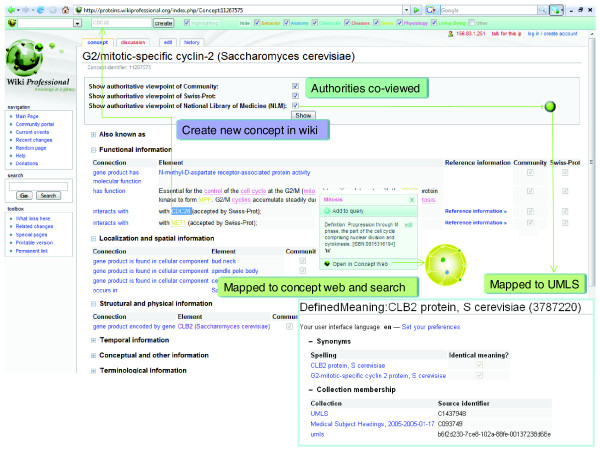
The WikiProteins Concept page of the *CLB2 *gene and its known formal synonyms (data from UniProtKB/Swiss-Prot as the authoritative database). Highlights are concepts recognized on the fly in the page that are linked to the corresponding Concept pages in the Wiki, to PubMed records, and to the concept space. Multiple terms selected in the page will create an AND query in external sources such as PubMed or a composite Knowlet with the selected concepts as source concepts (Figures 5-9). New co-occurrences on a given Wiki page due to edits by the community will be reported. Terms that represent concepts but are not recognized by the indexer can be added to the terminology system by selecting the terms in the text, starting a new Wiki page and defining the concept.

In OmegaWiki the name used for a specific meaning of a term is 'defined meaning'. In WikiProteins we call a defined meaning a 'concept' for consistency reasons with the concept space represented by the Knowlets. WikiProteins and OmegaWiki are both driven by a relational (MySQL) database that is linked to the concept space by on the fly indexing of all Wiki pages as soon as they are called. Concept recognition is presently done with the Peregrine indexer [14], coupled to a terminology system directly derived from OmegaWiki. We will invite colleagues running alternative indexing systems to co-index the full corpus of text in WikiProteins. This is likely to improve precision and recall of concepts to the maximum achievable with present best of breed text mining technologies. The WikiProteins terms mapping to known concepts are thus recognized in the Wiki text and other supported sites and automatically hyperlinked to their Knowlet in the concept space, their Wiki page and to their known occurrences in public literature databases. At the request of the user, all recognized concepts will be highlighted in the text and pop-ups allow concept-to-concept navigation within the Wiki, and related sites. It also allows easy construction of composite Knowlets from the selected concepts in a textual output (Figure 3).

Registered users can edit records from an authoritative database and change, correct or add data to that record. Upon saving the data, however, a new (copied) record in the community database is created, which can be viewed alongside the original data from the authoritative sources. Thus, the authority and the integrity of the participating authoritative sources are protected. Multiple threads of authorities and the community can be edited separately and can be converged again based on consensus. Several authoritative sources collaborating in this initiative have already indicated that they will formally recognize authors who have contributed significantly to the annotation and refinement of the information on certain concepts, such as proteins.

The first round of indexing and Knowlet creation has yielded over one million biomedical concepts in the Knowlet database, as well as the Knowlets of well over one million authors who currently have publications in PubMed. By matching concept Knowlets with author Knowlets it is now conceivable that those 'million minds' will annotate the few Knowlets most central to their expertise.

Combination of the Wiki and the Knowlet technologies enables the creation of an environment where scientists can combine their daily practice of knowledge discovery with close to real time collaborative comments and annotations. Edits that influence semantic associations will be reported automatically to other interested colleagues, as well as to the owners of the participating authoritative databases. The anticipation is that these resources will be amended, based on the community activities in the Wiki-environment.

Annotators of authoritative sources can use the information in the community database to facilitate their curation work and they can choose to record their activities in the community version as normal edits or comments. The community will judge the newly entered and amended data for credibility, as well as re-edit them where appropriate. This holds for updates in the authoritative source as well as for the community edits.

All edits can be viewed in the community history pages with real names of the editors associated. Thus, the level of expertise of the editor can be revealed easily: the person is a formal annotator, has many publications on the subject, is a formal guardian of this concept, and so on. Because of the formal registration, appropriate credits can also be given to active community annotators. The editor can also add peer reviewed references to the comments, to increase credibility and general acceptance of the edit. The expertise level of contributing community members can be judged from the publications associated with their name and the Knowlet based on their publications. Embryonic functionality review expert profiles will be available in the first launch of WikiProteins. Full social networking aspects, including several parameters relating to level of expertise and official 'guardianship' of certain concepts will be developed in close collaboration with a growing consortium of active users in order to serve the best practises developed.

Concepts for which no terms are present and defined in OmegaWiki are not identified by the Peregrine indexer and thus not highlighted in web pages. Registered users can manually select them in the page and start a new concept page in WikiProteins with one click. A definition of the selected expression will give it defined meaning status and unless the community rejects the entry, the term will soon be considered a valid concept. Each term added in WikiProteins will be synchronized with OmegaWiki, where translations and other terminological additions can be given. The Peregrine indexer will soon highlight newly added concepts, but they will be marked as 'under construction' for a given period of time. When text and references are added to the concept page, the Knowlet of the new concept can be created.

## User scenarios

### Community annotation

**Figure 4:**
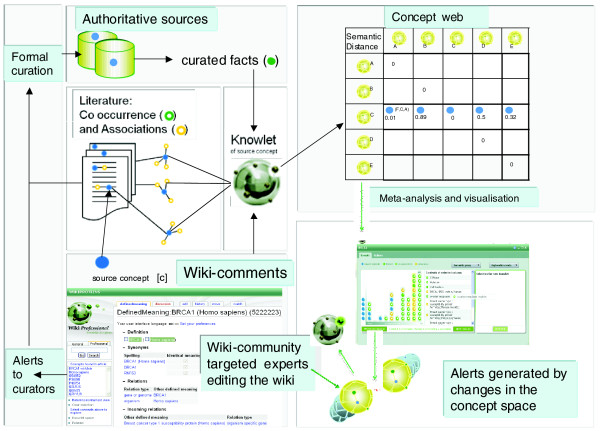
The basics of community annotation and semantic support. Once Knowlets have been created from authoritative sources and the indexed literature, a regular re-computing of the concept space with all changed semantic associations is performed. In case new co-occurrences, stated facts or significant associations emerge from the computational process, all experts that have expressed an interest in that part of the concept space will be alerted. Pre-constructed Knowlets for over one million authors have been created who currently have publications in PubMed. When they comment in the Wiki, their contributions will automatically be indexed and processed, forming an additional source for Knowlet enrichment alongside the classic literature and databases. UniProtKB/Swiss-Prot, GO Annotation, IntAct and UMLS have indicated that they wish to use the system as a source for accelerated annotation in their respective information resources.

The central goal of WikiProteins is community annotation of biomedical concepts and their interactions. The basic principle of community annotation is that computers and experts interact in an iterative process of mining and curation, as pictured in Figure 4. The various new technologies, terms and approaches adopted to enable this process will be described in more detail below, but first the basic principles of the approach are explained.

The biomedical literature contains pertinent 'facts', that is, statements of relationships between concepts that are generally considered to be scientifically 'accepted'. Each new article contains many repetitious factual statements, with references, along with a limited number of 'novel' facts. New facts will frequently also cause novel co-occurrences. As a consequence of removing factual redundancy, the number of unique facts (and thus the concept space) expands with only a fraction of the total number of sentences in the biomedical literature (Figure 1; see the 'Rationale and overview' section).

A growing subset of these relevant facts, such as the described functions of proteins, protein-protein interactions or protein-disease relationships, have already been annotated and curated in open access databases and ontologies, such as the UMLS and UniProtKB/Swiss-Prot, IntAct, and GO Annotation. These and other on-line resources have become indispensable tools for current biomedical research. However, the rate of growth of high throughput data and published information in the life sciences renders comprehensive and timely annotation of the literature for actual facts by any central team of experts an unachievable goal. Computer assistance in the annotation process is, therefore, urgently needed.

Recognizing concepts in free text is not trivial, not even for human readers, let alone for computers. The yeast protein CLB2 is an instructive example. The (incorrectly spelled) term 'Clb2', used as an example in [2], when typed into UniProtKB/Swiss-Prot, leads to 25 entries. One is the correct concept - the gene coding for G2/mitotic-specific cyclin-2 (see Figure 3 for its WikiProteins page) - but the incorrect synonym used by the original authors is not listed in the corresponding Swiss-Prot record, neither as a synonym of the corresponding gene name nor of its protein. But Clb2 is, for instance, also a synonym for emb-9, which encodes the Collagen alpha-1(IV) chain in *Caenorhabditis elegans*.

In the *Saccharomyces *Genome Database [15], the formal name of the gene is CLB2, and the synonym Clb2 is not listed; however, the query term Clb2 leads to the correct gene. A focused database like *Saccharomyces *Genome Database can let its internal search engine be case insensitive and find CLB2 based on the query term Clb2, but in a wider context, case insensitivity leads to aggravation of the ambiguity problem. For example, in PubMed, the query 'Clb2' delivers papers on dental self-etching primers such as 'Clearfil Liner Bond 2' [PMID: 9522695, 12601887], on the *Clb1 *gene in the fungal pathogen *Ustilago maydis *[PMID: 14679309] and on calcineurin B-like proteins, such as CLB1 in *Arabidopsis *[PMID: 14617077].

For computational meta-analysis this ambiguity is a severe limitation. In earlier microarray case studies we typically found that roughly 40% of all gene names in our lists have homonymy problems of some sort (unpublished data). Most of the re-writing rules to improve 'fuzzy' recall of gene and protein names have negative effects on precision and only marginal positive effects on recall [16]. Thus, non-standardized use of terms in the literature induces vast problems of homonymy and these are not easy to solve.

In WikiProteins, various algorithms have been implemented to keep the homonym problem to the minimum achievable with the current techniques for word sense disambiguation [17]. However, false positives for co-occurrence of two concepts in a sentence based on homonyms still happens occasionally and will be a disturbing factor in WikiProteins also. In contrast to 'read only' sources on the web, in WikiProteins, users are able to enrich the terminology system, thus improving concept recognition in future instances of indexing the same records.

In the natural language of standard scientific literature, the majority of simple facts have been described within one sentence, although in some cases a factual statement may be spread over multiple sentences. Attempting to mine these 'scrambled facts', in early case studies, only marginally increased the recall of actual facts and introduced many errors [18]. Attempts to mine multiple sentences and paragraphs in the broad biomedical literature for all individual instances of a unique factual statement have met with limited success and, in fact, may have very little added value for meta-analysis of the literature as a whole [1]. Unless the fact is very new, multiple instances of statements in sequential publications are only of use, from a computational point of view, to increase the likelihood that the statement is a consolidated fact. For well established facts one does not need to find the very last instance of the factual statement in all papers to be able to present the fact correctly in an ontological format such as the Knowlet. We have chosen, therefore, to analyse texts at the sentence level and accept the trade off with optimal recall of individual statements.

For Knowlet construction the number of sentences found affects the value of the C parameter (Figure 1), but in many instances where the C parameter is positive, there is either factual or associative information involved in the computation of the semantic association. Logical co-occurrences suggested by the mining technologies as 'potential facts' are actively presented to registered experts for community annotation. Where possible, confirmation of factual status should be reported in the Wiki with references to sentences in the peer reviewed literature as supporting evidence.

An additional major limitation of classic text mining approaches is that much of the relevant text is securely behind the firewalls of publishers and is not easily accessible for automated indexing. This is another reason why it is not possible to exclusively rely on computational text mining as a definitive source for facts. In fact, roughly 60% of protein-protein interactions mined from Swiss-Prot and IntAct cannot be found co-occurring in a PubMed sentence or even an abstract (H van Haagen and A Botelho-Bovo, in preparation). This should not be considered surprising, as much of the information leading to those annotations came from full text articles, and within these from tables and figures, many of which are not suited for computer indexing. Thus, a large, intrinsically motivated community of experts is needed to accelerate the curation and annotation process of mined 'potential facts'. Copying of relevant sentences from full text literature with reference to the original article is one of the goals of WikiProteins. Easy tools for recognition of new co-occurrences (that is, not occurring in PubMed), but only in full text articles, are under development. Digital object identifiers of the underpinning articles can be downloaded in the Wiki environment to support factual statements by registered scientists. As more new relationships are validated, this approach may lead to collaborative knowledge discovery. This iterative human-machine interaction is a perceived central aspect of community annotation.

Based partly on the concerns described above, several attempts have already been made to involve the scientific community in annotation [19-22], but so far with limited success. We postulate that this slow adoption of collaboration via web services is due both to the perception of immature applications for annotation and to the fact that distributed annotation is widely perceived by busy scientists as a service to their colleagues only, and much less as a crucial activity for their own research work with immediate positive returns. However, community annotation aims to create and support stable and growing communities of interest around certain concepts, such as genes/proteins, pathways, diseases and drugs, with incentives for keeping information fully up to date.

Several colleagues have recently communicated a spontaneously growing activity in the current Wikipedia environment to annotate protein and RNA related pages (A Bateman, personal communication). WikiProteins is automatically linked to such community annotations in Wikipedia through the on the fly concept recognition. More direct mapping approaches are being developed. This hyper-linking allows annotations in both environments to be captured in the concept space.

It should be emphasized that editing in Wikipedia is not restricted to traceable registered users and that Wikipedia is meant to represent a neutral point of view. WikiProteins is complementary in that it provides a more structured environment where more original data and scientific debate can be accommodated, as well as a direct collaboration with authoritative sources. We anticipate, therefore, a co-existence and complementary role for Wikipedia and WikiProteins.

### Knowledge browsing

A second user scenario is the use of WikiProteins to browse quickly through the concept space for interesting relationships.

To demonstrate the current status of the Knowlet based system we will use the following sentence from PMID 15920482: "Mitotic cyclin (Clb2)-bound Cdc28 (Cdk1 homolog) directly phosphorylated Swe1 and this modification served as a priming step to promote subsequent Cdc5-dependent Swe1 hyperphosphorylation and degradation." Jensen *et al *[2] discussed this example in their review and made the following statement regarding this sentence: "Current *ad hoc *IR systems are not able to retrieve our example sentence when they are given the query 'yeast cell cycle'. Instead, this could be achieved by realizing that 'yeast' is a synonym for *S. cerevisiae*, that 'cell cycle' is a Gene Ontology term and that the word Cdc28 refers to a *S. cerevisiae *protein, and finally, by looking up the gene ontology terms that relate to Cdc28 to connect it to the yeast cell cycle. Although this will not be easy, we see this form of query expansion as the next logical step for *ad hoc *IR." WikiProteins is not to be perceived as an information retrieval (IR) system, but it is illustrated below that the concept space may nevertheless serve this stated need.

First, when the full abstract [15920482] is put into the concept recognition window, the ambiguity in the language becomes quite apparent. '*S. cerevisiae*' is called 'budding yeast' in the title and the only protein mentioned there is 'Swe1/Wee1'. Furthermore, the authors of this abstract have used several constructs that make text mining difficult as they enter conjugate terms such as 'mitotic cyclin (Clb2)-bound Cdc28 (Cdk1 homolog)', 'Clb2-Cdc28', 'Clb2-Cdc28-phosphorylated Swe1', 'Cdc28/Cdk1', and 'Cdc5/Polo'. Many difficulties are introduced by using non-preferred names for genes and proteins and, particularly, by using dashes and slashes that are not parts of the gene symbol, but are simply separators for conjugated terms. The text further mentions that Wee1 is a protein kinase.

Despite this high degree of ambiguity in the terminology in the test abstract 15920482, the Peregrine indexer recognizes several meaningful concepts in the abstract: the proteins Serine/threonine protein kinase; Wee1 like protein kinase; Protein arginine N-methyltransferase HSL7: Cell division control protein 2, based on the synonyms Cdk1 and Cdc28; the concepts bud neck, and mitotic entry; the GO term cyclin-dependent protein kinase regulator activity; Polo-Box domain, phosphorylation; and the organism *Saccharomyces*. A click on the PMID 15920482 will lead to the concept web-linked version of the abstract.

**Figure 5:**
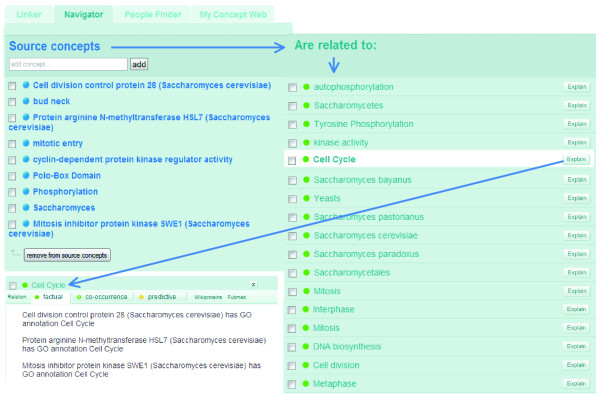
A total of 26 concepts are recognized by the Peregrine tagger (2007) in abstract 15920482 from PubMed (see first column). The associated concepts in the composite Knowlet of these concepts include those that are expected, as discussed in the main text.

Notwithstanding the severe problems in this abstract for automated indexers due to ambiguity, the composite Knowlet that was automatically created from this abstract has the following concepts in the histogram (Figure 5): cell division, cell cycle, *Saccharomycetes*,, kinase activity, yeasts and mitosis. From this first case study it can be concluded, therefore, that the Knowlet of this abstract associates its content very strongly with the query 'yeast' and 'cell cycle', partly due to our thesaurus-based mapping of budding yeast to *Saccharomyces*. Further improvement of protein recognition and recognition in highly ambiguous text will dramatically improve this output.

When the selected sentence is taken by itself for indexing, only one of the proteins is correctly recognized by the indexer. Nevertheless 'cell cycle' and 'mitosis' are central concepts in the resulting Knowlet. The connection to 'yeast' disappears, which is due to the poor species-specific recognition of proteins in the sentence and the absence of a reference to yeast in the sentence itself.

As a second example, the respective proteins from the case study sentence were mapped with the WikiProteins dictionary look up to the following concepts with the preferred terms: Clb2 = G2/mitotic-specific cyclin-2 (S. cerevisiae) Swiss-Prot P24869; Cdc28 = Cell division control protein 28 (S. cerevisiae) Swiss-Prot P00546; Cdk1 = homolog of Cdc28; Swe1 = Mitosis inhibitor protein kinase SWE1 (S. cerevisiae) Swiss-Prot P32944; Cdc5 = Cell cycle serine/threonine-protein kinase CDC5/MSD2 (S. cerevisiae) Swiss-Prot P32562

**Figure 6:**
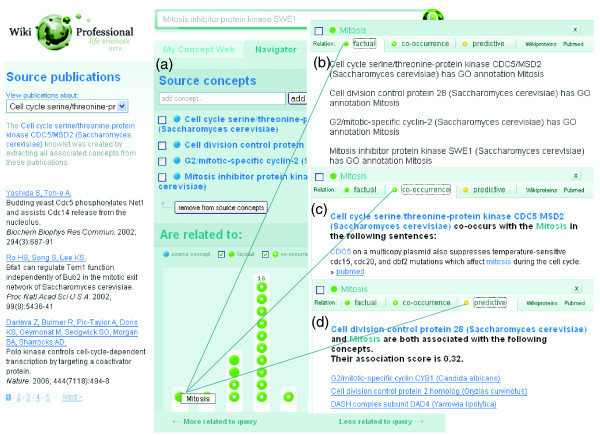
The Knowlet-based connections of four yeast proteins. **(a) **The composite Knowlet of the four yeast proteins as indicated in the text. **(b) **When the (factually connected) concept 'mitosis' is selected for explanation in the Knowlet, the factual association appears to be based on GO annotations. **(c) **Multiple co-occurrences are also found with more than one source concept including *S. cerevisiae *and CDC28. **(d) **In addition, there are multiple concepts that indirectly connect the source concepts with cell division. This means that the original example sentence used for this case study would have been repeatedly retrieved as relevant in the 'explain' window, supporting by co-occurrence the semantic association between the proteins involved.

The Knowlets of these proteins were aggregated in the concept space. The system creates the Knowlet-output shown in Figure 6. In discovery mode (Figure 6a; preference for co-occurrences and associations over facts), the closest factually associated concept in the graph is 'mitosis'. The strong semantic association between 'mitosis' and the four source concepts is mainly caused by factual relations (GO annotation) of all four source proteins (Figure 6b). In addition, there are co-occurrences (Figure 6c), and, finally, there are many associative concepts (Figure 6d). The same Knowlet, presented in background mode, shows the concept 'cell cycle' prominently present for mainly the same reasons.

The main conclusion from this particular example is that the future aim to associate the selected sentence with the concepts 'yeast' and 'cell cycle' is, in fact, not primarily hampered by the fact that the two terms or their synonyms are not mentioned in the sentence. With this level of language complexity and ambiguity, the problem is more related to the lack of adequate computer-recognition of (wrongly spelled) terms (see also the 'Rationale and overview' section). Methods that take context and factual knowledge from databases into account, like the one described here, will relate the case study sentence to the desired terms.

It should be emphasized again that creating a factual and associated concept space around 'yeast cell cycle' with appropriate links to supporting sentences for each edge in the network is a more useful approach to knowledge discovery than the retrieval of a single sentence.

### Collaborative knowledge discovery

The third scenario serves to demonstrate the potential for knowledge discovery using the WikiProteins resource and community annotation.

**Figure 7:**
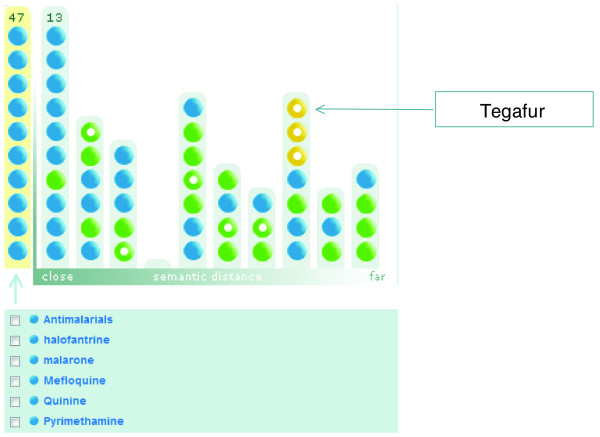
Screenshot of the broad concept space of the concept 'antimalarials' and 46 actual antimalarial drugs. The closest, non-co-occurring drug is tegafur, which was explored further (see main text).

When the composite Knowlet of the concept 'antimalarials' and 46 known antimalarial drugs is viewed in discovery mode with the semantic filter on 'chemicals' only, there are three yellow rings, which represent concepts associated with this space only by indirect association (Figure 7). These concepts are 'mdr gene/protein plasmodium', 'dehydrofolate reductase' and the drug 'tegafur'. The first two concepts are logical associations with malaria. Tegafur is not obvious and does not have any co-occurrence in PubMed with 'malaria', 'plasmodium', or 'antimalarials' as checked by a regular PubMed search on 28 December 2007.

**Figure 8:**
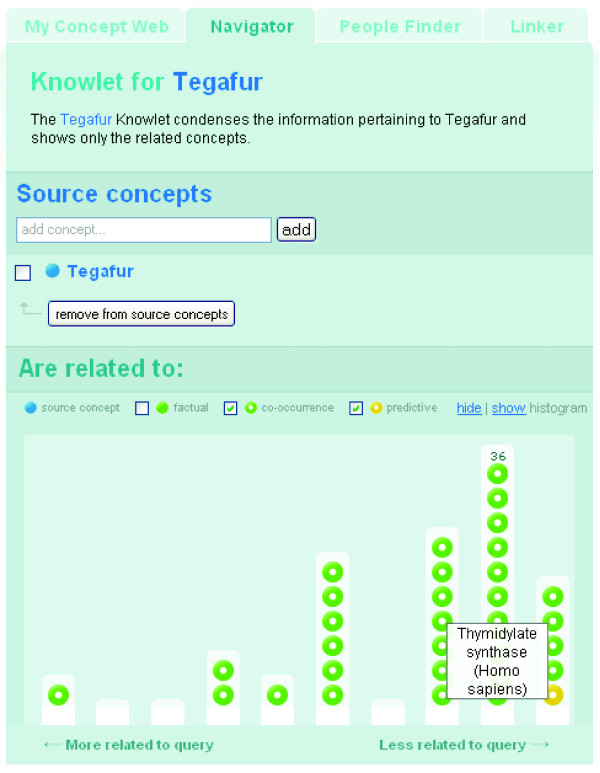
The Knowlet created with the source concept 'tegafur' with TS as a closely associated concept.

The interest of a researcher may be sparked by the enzyme and cell division related concepts in the Knowlet of the anti-neoplastic drug tegafur and this may lead to the construction of the Knowlet depicted in Figure 8, where the source concept represents 'tegafur'. The most highly associated enzyme in this Knowlet is 'thymidylate synthase' (TS).

When PubMed was consulted, out of 2,991 abstracts on tegafur, several mentioned the enzyme as a target for the drug. An 'AND' query with 'malaria' and TS yields 55 abstracts among which is the article 'Evaluation of the activities of pyrimethamine analogs against *Plasmodium vivax *and *Plasmodium falciparum *dihydrofolate reductase-thymidylate synthase (TS) using in vitro enzyme inhibition and bacterial complementation assays' by Bunyarataphan *et al*. [16954316]. This abstract contains the sentence: "The 50% inhibitory concentrations derived from PvDHFR-TS-dependent bacteria were correlated with their corresponding inhibition constants (Ki) from an enzyme inhibition assay, pointing to the likelihood that the potent enzyme inhibitors will also have potent anti-malarial activities." The procedure described has correctly revealed an indirect association in the concept space that could indicate that tegafur is a candidate anti-malarial drug.

**Figure 9:**
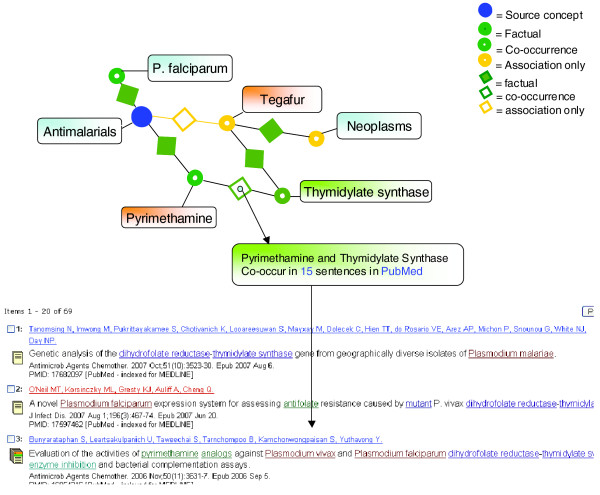
An artist's impression of the all-to-all semantic association matching of selected concepts followed by two-dimensional visualization. Several techniques for visualization of the concept space by standard techniques such as multi-dimensional scaling are currently under development.

When the connections in the concept space around antimalarials and tegafur are explored further, it becomes immediately obvious how logical it would be to reason that tegafur might indeed inhibit growth of malaria parasites, at least *in vitro *(Figure 9) Obviously, multiple reasons could exist for why the compound may not work, including physical reasons, such as prevention of entrance into erythrocytes based on the molecular size of tegafur. It is beyond the scope of this paper to investigate these associations any further, but it serves as an example of the principle of Knowlet-based discovery.

## Final considerations and future outlook

### Collaborative knowledge discovery and alerts as a major incentive

The system presented here is the very first start of an environment in which social networks of scientists with a common interest can collaborate with the aim of making the representation of 'their' part of the concept space more accurate, which has the immediate potential to uncover and alert them to previously evasive relationships in the process. For instance, in the hypothetical situation that it was recently discovered that tegafur inhibits the function of TS and that the first paper about this association with the enzyme was indexed in WikiProteins, experts who had saved an 'antimalarials' Knowlet in their system would be alerted, because the concept 'tegafur' would have entered the concept space of that Knowlet.

Moreover, the concept space of any drug or gene list will naturally contain a number of associated diseases that may be interesting for further study. In the light of the recent commentary by Chong and Sullivan [23], this could be approached systematically to generate candidate diseases for drugs in a more sophisticated way than has been done previously [24,25].

### Wikiprofessional, a crucial future element of community annotation

The concept profiles of more than one million individual authors from PubMed have already been pre-constructed. Using disambiguation algorithms as partly described before [26], as many papers as possible for each unique author have been automatically collected. With these papers, an 'Author concept profile' has been constructed and, subsequently, an Author Knowlet. These will be augmented by a highly curated database from Latin America, CV Lattes [27], which contains considerable overlap with the PubMed Author collection, but will also enrich the system with authors that cannot be easily found via PubMed. The concept space of experts enables WikiProteins and any other conceived professional Wiki to assign any fact, potential fact or potentially interesting associative concept combination to a natural community of interest, namely to that group of authors that share most of the concepts in question in their personal publication Knowlet. Thereby, the system can target alerts to specific and knowledgeable groups of experts. Even if only a small percentage of these experts reacts by commenting on a suggested fact or an interesting new association beyond direct co-occurrence, the facts can become 'community reviewed' and approved by a list of experts until they can be accepted as 'factual'. By participating in the system with an author approved Knowlet, scientists can contribute to the collection and approval of facts from the literature, but they can also confirm 'factuality' of (C+) or even only (A+) relationships in their expertise area, which will influence the overall concept space.

The first release of WikiProteins contains an embryonic version of what is intended to be developed into a fully functional WikiProfessionals in 2008 and beyond. Users are able to review their pre-constructed (recent) publication list and create their Knowlet before registration. With an increasing number of authors having curated their own Knowlet(s) in the system, creating communities of expertise and indicating their availability for comments and peer review, instant messaging and web conferencing will become available in the system. The system also bears great potential to create a unique author ID, a stated need in all publishing environments.

### External indexers, databases and ontologies

The data contained in the open content Wiki-environment are open for any research group to be analysed with their own taggers, indexers and text analysis algorithms. Generating additional data by such efforts can capture the relevant factual, co-occurrence and associative relationships in the publicly available system. In addition, all owners of authoritative databases or biomedical ontologies are invited to connect to the Wiki system to enable community-assisted enrichment of their resource. The National Center for Biomedical Ontology (NCBO) will facilitate these efforts.

## Conclusion

A consortium has been formed to construct the first public, interactive web service providing both factually and potentially associated concepts, including proteins and genes. Based on frequent meta-analysis of biomedical ontologies, published databases, the Wiki and new sentences collected by the community from the broad scientific literature, the system will actively push new or suggested associations to experts for review and annotation in a Wiki-environment. The resulting community annotation layer is provided in addition to authoritative sources, not as a replacement. It is freely available to all interested parties, and special provisions are made for the participation of colleagues from developing regions.

Curated sources such as UMLS, UniProtKB/Swiss-Prot, IntAct and GO will mine the community layer for material to improve the quality of the records in their own platforms. It is believed that there is room for a persistent co-existence of an editable (Wiki-) layer of provisional annotation along with one or more sanctioned layers of 'established knowledge', defined here as 'authoritative sources'. Additionally, complementary resources can be linked to the system.

It is anticipated that WikiProteins will accelerate computationally assisted, collaborative knowledge discovery in conjunction with annotation of factual information from the literature, from rough data and from direct experimental evidence. The various filters on the concept space reduce the risk for spurious co-occurrences and associations to a minimum, but they also allow users to explore less obvious connections if so desired.

The indexing so far has revealed that the 'million minds' approach can be taken quite literally and the consortium invites the currently active scientific community to annotate minimally one Knowlet on which they are an expert. In doing so, they may also include sentences from articles that are not available in open access and are, therefore, inaccessible to public text mining, with proper reference to the original article.

### Potential further developments

In terms of content presently represented in the Wiki and the externally indexed resources, the prototypic version of WikiProteins is only a start. This automatically translates to the richness and the quality of the current Knowlets. The consortium intends to add progressively more authoritative resources to the community annotation system, but in the Wiki-spirit the community is encouraged to bring in new technologies and content into this open access environment. In principle, all high quality resources describing interactions between biologically meaningful concepts could greatly benefit from inclusion in this environment, and their integrity and data-ownership is guaranteed via the authoritative source protection. Although all data in the community database of the Wiki are subject to the GPL-CC-by license (freely downloadable and re-usable), individual authoritative databases retain their own copyright and can put restrictions on the use of their original data supplied to the Wiki for Professionals environment.

The consortium can assist new candidate authoritative sources with technical advice on the development of dedicated import scripts of (selected) data from the source database. Upper ontologies will secure data consistency. Updating of the individual authoritative source will take place through dedicated update scripts for each source, which are anticipated to remain the prime responsibility of the respective database owners. The consortium will also develop technology to allow a more 'federated' approach where original data from authoritative sources are no longer physically imported into the Wiki database but are 'Wikified' in the founder sites.

Once widely used and augmented, this resource could become an open, yet quality assured and comprehensive, environment for collaborative reference and knowledge discovery.

## Abbreviations

GO, Gene Ontology; TS, thymidylate synthase; UMLS, Unified Medical Language System.

## Authors' contributions

BM, CC, EvM, MW, AM, NB, EM and PJR were actively involved in the design and testing of the WikiProfessional system. JdD, G-JvO, KB and MM provided scientific guidance during the development process. MC, HH, AP, RP, SL, MA and ABa provided essential database content as well as scientific and practical guidance for the project. ABe and WM provided strategic guidance and the major part of the funding for the development of WikiProfessional. BM, JW and GM conceived of the original idea of coupling the relational Wikidata software to the Knowlet space, which later developed into the full design of WikiProfessional with the entire team.

## Additional data files

The following additional data are available with the online version of this paper. Additional data file 1 provides a more detailed technical description of the construction of Knowlets and the Wiki system.

## Supplementary Material

Additional data file 1 Detailed technical description of the construction of Knowlets and the Wiki system. Click here for file
