# Supplementary material for: Calling on a million minds for community annotation in WikiProteins
Source: Genome Biol. 2008 May 28;9(5):R89. doi: 10.1186/gb-2008-9-5-r89 (PMC2441475; doi:10.1186/gb-2008-9-5-r89)
Supplement: Additional data file 1 — Detailed technical description of the construction of Knowlets and the Wiki system. [file gb-2008-9-5-r89-S1.rtf]

Section 3 (Addtional Data File)  

Supplementary Description of the system 

Development assumptions adopted for the Knowlet and the WikiProteins platform.

1.	To assist their discovery process, scientists are interested initially in already established facts in databases and the literature.
2.	These facts need to be of the highest possible 'confidence', preferably manually curated by multiple experts.
3.	Most pertinent facts can be captured in (combinations of) triplets of the format <concept A> <relation> <concept B>.
4.	Repetition of facts is of great value for the readability of individual papers, but the fact itself is a single unit of computable information, and needs no repetition.
5.	There is an intuitive relationship between the level of repetition of facts in the raw literature and the likelihood that the fact is 'true', but even multiple repetitions do not guarantee that a fact is really true.
6.	Beyond a (variable) threshold, further repetition of a fact does not increase the likelihood that the factual statement is true.
7.	Once a dependency triplet is found, establishing it as a fact by human expert curation is more valuable than finding multiple instances of the same fact by sophisticated text mining in the literature per se, although the latter still has added-value.
8.	If previously established 'facts' appear to be wrong or are nuanced, the scientific community should be aware of that. A crucial feature of on-line publication is that such facts can be retrospectively marked as erroneous or 'changed' in older literature. This is hardly happening today.
9.	Scientific Knowledge, especially as far as it is generated in the public domain should be freely accessible for all concerned [28]
10.	Therefore, any system reproducing facts from the public literature should be Open Access, especially a system that involves annotation by the broad scientific community.
11.	The system should be open to the application of multiple text mining tools and approaches.

Detailed description of the Knowlet principle 
(overview in figure 2, main article)

The Knowlet approach enables the combined description of multiple (currently three) main categories of relationships: Factual (F), Co-occurrence (C) and Associative (A). All three will be separately described below. In the near future, the addition of sequence similarity data and co-expression data for genes is foreseen (see figure 2.)

Facts (F)

We here define 'facts' as the simplest statements of the structure <concept><relation><concept>. This would comprise a whole range of relationships from 'is a' relationships to active biological interactions: <Protein A> <inhibits> <Protein B> or GO annotations such as <protein> <has GO annotation> <GO term>. Typical RDF triplets in ontology's, but also relationships as described for the Sequence Ontology proposed by Eilbeck et al. [29], can be supported as can any other direct relationship between two concepts. These factual statements can form the basis of much broader biological ontologies. 
For the initial factual Knowlet database construction, the UMLS (UMLS 2006AD version, which contains 1,029,091 concepts) and the UniProtKB/Swiss-Prot database (version 23 Jan 2007) have been used, which at that point in time contained 254,609 proteins.  Not all potential triplets contained in the UMLS have been imported but the system was restricted to mining those relationships potentially having a direct impact on biological discovery. Semantic groups for which it was attempted to complete the full Knowlet creation process are: 'Anatomy', 'Chemicals', 'Diseases', 'Organisms', 'Proteins' 'Behaviour' and 'Others'. 
The UMLS records containing concepts of the semantic type 'organism specific protein' were mapped to the protein entries in UniProtKB/Swiss-Prot to the extent possible by computational alignment. Additionally, the Gene Ontology terms and protein interaction pairs from IntAct that pertain to UniProtKB/Swiss-Prot records were utilized and mapped to the respective proteins. After partial disambiguation of expressions by means of the Biosemantics Genelist thesaurus which is optimized for gene/protein name recognition in text [30], the authoritative databases included so far have been mined for factual information. Whenever a source concept has minimally one factual (curated) relationship with a target concept, that target concept will be shown in its Knowlet as a solid green ball (see figure 1). If that ball is clicked for 'explain', the 'factual' tab will show the nature of the mined relationship. Some examples of factual relationships that may be found under that tab in the Knowlet of the four example yeast proteins are:

·	Cell cycle serine/threonine-protein kinase CDC5/MSD2 (Saccharomyces cerevisiae) has GO annotation Mitosis
·	Cell division control protein 28 (Saccharomyces cerevisiae) has GO annotation Mitosis
·	G2/mitotic-specific cyclin-2 (Saccharomyces cerevisiae) has GO annotation Mitosis
·	Mitosis inhibitor protein kinase SWE1 (Saccharomyces cerevisiae) has GO annotation Mitosis 

As the concept Mitosis has a mined factual relationship with all four source concepts, it will be depicted as a green ball as explained in section 2, main paper.

Facts from protein databases on interactions may also be part of the factual tab:

·	Mitosis inhibitor protein kinase SWE1 (Saccharomyces cerevisiae) interacts with Probable serine/threonine-protein kinase KCC4 (Saccharomyces cerevisiae) 

From IntAct
 
One might also find more terminologically based relationships from UMLS

·	Muscular Dystrophy, Duchenne is a form of Muscular Dystrophy
·	Muscular Dystrophy, Duchenne is possibly synonymous to Muscular Dystrophies
   
So far, factual relationships only have been mined from the currently participating authoritative sources. Many more have been defined that can be mined from the current group, but new databases soon to come in to the system, e.g., the Genetic Association Database [10], will enrich the Concept Space with thousands of additional factual relationships. If a fact is restated by several databases, for instance an interaction recorded in IntAct as well as in Swiss-Prot, the factual parameter may be given a higher value.
The Concept Space will be progressively complemented with more ontologies and databases and the community is invited to pinpoint data sets that are of high quality and common value. 
 
Facts 'without co-occurrence'

One might intuitively expect that in most cases where (F) is positive, there must be a sentence, or a set of sentences somewhere in the biomedical literature at large containing the two relevant concepts.  If such evidence is not found in PubMed, it is apparently to be found only in the full text article or in resources currently not covered by PubMed. This latter situation appears to be frequent, especially for protein pairs from IntAct and Swiss-Prot. A separate paper is in preparation which shows how frequently facts cannot be recovered from the public literature with a normal 'AND' query (Van Haagen and Botelho-Bovo pers. comm.). 
In fact many gene- and protein lists from genomics and proteomics studies are being published in extended tables and supplementary files that may or may not be in readable format by automated indexing of these full text papers. The lack of PubMed co-occurrences for already curated facts is not a problem for the WikiProteins system as the factual statements from the explain window refer to the authoritative source.
Soon a PubMed service will be provided in the WikiProfessional environment yielding indirect links between concepts whenever the PubMed search engine does not retrieve any abstracts based on co-occurrences.

Co-occurrences

One immediate aim of WikiProteins is the collection and Community Annotation of individual triplets representing pertinent facts. As stated before, most facts are at least once comprised in a single sentence, but are concept combinations, even if they are selected for potentially meaningful components of a triplet, a good source for community annotation of facts?
Early Alpha testing of WikiProteins suggests that the vast majority of 'green rings' in the Knowlet based on co-occurrence with the source concept, actually do represent factual relationships of some sort. It is intuitive that there are many facts that are true and published, but are not contained in a curated database, either because the database team has not yet covered it because there is simply no database (as of yet) dedicated to that class of facts, or because the fact is considered to be "general knowledge".
The 'co-occurrence factor' between any given pair of concepts has been designated (C). A relationship between a source concept and a target concept is C+, when minimally one sentence containing both concepts has been found in the indexed resources. The value of the (C) parameter is either the number of sentences found per se, or a calculated value corrected for the a priori likelihood that two concepts appear together. Presently, the co-occurrence factor is based on over 112 million separated sentences from PubMed abstracts published after 1996. Progressively algorithms will be implemented to predict the factual information from co-occurrence, both in abstracts and in full text literature with increasing precision.
Concepts are always treated as pairs in a sentence. For example, in the screenshot below (fig. 1) there are many other co-occurrences to be found in the displayed sentences than just 'CDK1' and 'G2 Phase'. These sentences will therefore appear in the explain windows of all other concept pairs contained in that sentence. In all cases, only the two concepts under study are highlighted. 


In many cases it is fairly easy to find a sentence stating a relatively simple factual relationship between the concepts under study. In this example the sentence 'to copy to the Wiki' may be: 

·	In all eukaryotes, entry into mitosis from G2 phase is initiated by a complex of the cdc2 kinase and a B-type cyclin. 

Experienced WikiProteins users may take the time to enter this statement in the correct Swiss-Prot syntax via the semi-structured editing options in the Wiki, and list the sentence and its PMID as a reference. However, if a novice user decides to just copy and paste this sentence to the Talk page of the Wiki it will also generate co-occurrences and alerts. 
In the near future, it is intended to provide a user-friendly system for voting on the validity of triplets pre-filled with potential facts as calculated by computational techniques. In the editing page of the relational Wiki, the typical relation types constituting triplets for certain combinations of concepts are supported in drop down menus for semi-structured data entry. All Wiki entries are indexed after the user has saved them and new co-occurrences will be captured and reported to interested experts.

Indirect Associations

A third category of relationships (A) supported in the system is the contextual, association between two distinct concepts which can reveal complex, indirect relationships and thus lead to actual knowledge discovery.
In case neither (F) nor (C) is positive for a given pair of concepts, there may still be circumstantial evidence for a meaningful relationship between the concepts, even if the association is only implicit. Consortium members and others have recently published on such 'hidden connections' [30-33]  
Associative connections are captured in the Knowlet as a third parameter, (A). This parameter is actually expected to represent the most interesting aspect of the Knowlet. Obviously, as facts are moved from (C+) and (F-) to (F+), the database becomes more factually solidified. However, bringing a concept combination from (F-), (C-) and (A+) to (F+) will either yield new co-occurrences and facts missed so far or, more importantly, may in fact be part of the online knowledge discovery process by in silico reasoning and potentially related laboratory experiments to confirm literature based hypotheses.
Target concepts only related to a source concept by association are presented in the Knowlet graphic as yellow rings.

The indirect association between two concepts is calculated based upon the matching of their individual Concept Profiles. A Concept Profile is constructed as follows (see reference [33] for details): For each concept (mostly entities) in the database, a number of PMIDs have been retrieved in which that specific concept has a significant incidence. In this case high precision was favoured, at the expense of recall. The goal is namely to construct a list of concepts from minimally one but up to 250 selected abstracts that are 'about' that source concept. The ranked concept lists were constructed by terminology-based concept-indexing of the entire abstract, followed by weighted aggregation with described algorithms into one list of concepts. The concepts in this list exhibit a high association with the source concept. These lists can now be expressed as vectors in multidimensional space and the associative score (currently the inner product is used) for each of the vector pairs is calculated. This associative score is recorded as a value between 0 and 1 in the (A) category of the Knowlet. In other words, even for those concepts between which the (F) and the (C) parameters are negative, a positive association score in (A) beyond a statistically defined threshold may indicate that there is significant conceptual overlap in their respective Concept Profiles to suggest an as yet non-explicit relationship. Recently it has been shown that a high conceptual overlap in two profiles can be predictive for an actual association hitherto undiscovered [32]. Thresholds can be calculated by comparing the distribution concept profile matches of non-related concepts of certain semantic types with those that are known to interact: for instance all proteins that are not known to interact with those that are known to interact in Swiss-Prot and IntAct [Van Haagen and Botelho Bovo in preparation].

Knowledge alerts and Knowledge discovery
 
The research described above has indicated that the literature contains a wealth of implicit knowledge that is too complex and dispersed for the reader to synthesize without computer assistance. The potential to discover these 'hidden associations' goes far beyond classical text mining. Obviously, text mining is needed to identify concepts and potential facts in literature, but even the co-occurrence per concept pair in more than 110 million PubMed sentences or the more sophisticated mining of 'factual dependency triplets' can only reveal stated, explicit, facts. Multi-step coupling of mined A>B and B>C into A>B>C relationships (basically reasoning in graphs) is an interesting approach.  More importantly, it is evident that the potential of the system to derive associative information from the literature beyond what has been explicitly stated is a crucial factor for success of any Community Annotation effort, as it supports scientists in their day-to-day pursuit of scientific breakthroughs. An example of this approach using the Concept Space is given in section 2.
A Wiki environment supported by authoritative sources is important as source information for this kind of knowledge discovery. It is intuitive that the more facts are available to a discovery system in unambiguous, computer readable format, the better algorithms for knowledge discovery can unearth new associations. Therefore an alert system was implemented to alert registered scientists when a concept enters the space of one of the stored interest Knowlets on their Wiki desktop. A pop-up shows the reason for the alert and a natural community peer review of the textual contribution that led to the repositioning of concepts in the Concept Space is anticipated.

The Wiki component

The Community Annotation component of the system maximizes recent Wiki software improvements by creating an environment where scientists can combine online knowledge discovery and data verification with annotation. This new approach is based on several recent developments; the high quality of Wikipedia records [34,35], the advent of the Open Access movement and the automatic semantic enrichment and the disambiguation of concepts, including gene and protein symbols in free text. [36]
The major extension developed to complement the established Mediawiki software powering Wikipedia, is the Wikidata software. This software allows Supervised Community Management of a relational database system. The system contains versioning features as well as registration modules since it is anticipated that this software will be used in a professional, Open Access environment by registered experts. The Knowlets form the basis of the semantic support of all Wiki's connected via the underlying terminology system OmegaWiki, which is a relational Wiki in and of itself.

Starting from Authoritative Sources

The Wiki element of the system is not starting as a blank slate as did Wikipedia. An important adaptation to facilitate collaboration is that the software supports the import of what we have designated as 'Authoritative databases'. Currently, selected records from the Unified Medical Language System (UMLS), UniProtKB/Swiss-Prot, Gene Ontology (GO) and IntAct have been jointly imported. 
Progressive inclusion of Reactome and all relevant ontologies in the OBO repository and beyond is provisionally planned. Such Authoritative sources will be treated as separate databases and as such they are 'untouchable' (read only) in the Wiki environment but they will provide the basis for gathering additional information (Figure 3, main text). Community annotation will be supplementary, and performed on copies of the original records, so that the latest, most mature stage of the community data can always be combined with the latest version of the authoritative source. The colleagues responsible for the authoritative sources intend to review the community annotations, and possibly incorporate formal versions of selected community annotations into their databases [37]. Upon the next regular update of these respective sources, curated community annotations may therefore be part of the authoritative database records. To avoid diversion beyond a healthy discussion and annotation level annotators of the authoritative database will be encouraged to add to the Community record that they have reviewed and used or why the information has been disregarded. They can also contact the Wiki-contributors via the User Page and e-mail facility in the Wiki or directly. Community Annotation will be attributable in the Wiki to the (group of) registered experts that have contributed to the editing and it is for each individual professional user to judge whether the community view is of added value (and can be trusted) before the annotation has been approved by uptake in an authoritative source.
Authoritative sources may recognize the contributions by Wiki-registered experts.
It is therefore encouraged that authors sign up and approve their automatically suggested list of recent publications before using the system.
The need for Open Source software to allow community editing of data and information of a more structured character than can be currently supported by Wikipedia is apparent. Other initiatives such as the alpha version of Freebase [38] show the arising need to provide the community with information that is much more structured than just manually hyperlinked flat pages. The Semantic Mediawiki approach [39], much like Freebase is an attempt to bring underlying syntax into flat text to enable more structural approaches (generate lists and tables on the fly for example). At this point we feel that the Wikidata approach is needed to support essential features for WikiProteins and other Wiki's for professionals, such as the support of integrated authoritative data sets and their separate management as well as the connected semantic support. However, it might very well prove possible to combine features of Freebase, Semantic Mediawiki and Wikidata into future applications covering an ever expanding need for community annotation.
